# Digitization and Active Aging: How Digital Finance Shapes the Mental Health of Empty-Nest Older Individuals

**DOI:** 10.3390/healthcare13172189

**Published:** 2025-09-01

**Authors:** Qian Luo, Haomiao Zhang, Weike Zhang, Dijia Shi

**Affiliations:** School of Public Administration, Sichuan University, Chengdu 610065, China; 2023325010011@stu.scu.edu.cn (Q.L.); zhanghaomiao@scu.edu.cn (H.Z.); 2023141090047@stu.scu.edu.cn (D.S.)

**Keywords:** digital finance, depression, empty-nest older individuals, active aging

## Abstract

**Background**: In the social context characterized by digitization and population aging, empty-nest older individuals have emerged as a particularly vulnerable group. This study examines the impact of digital finance on the mental health of empty-nest older individuals using data from the China Health and Retirement Longitudinal Study (CHARLS) spanning 2011 to 2018 and investigates its underlying mechanisms through the three dimensions of active aging. **Results**: The findings suggest that digital finance is associated with reduced likelihood and degree of depression among empty-nest older individuals. The beneficial effect of digital finance on depression is more pronounced among empty-nest older individuals with lower educational attainment, without supplementary pensions, and residing in rural areas. Furthermore, mechanism analysis shows that digital finance relates to better health outcomes, greater security, and increased social participation among empty-nest older individuals. **Conclusions**: These findings underscore the potential of digital finance to facilitate active aging among empty-nest older individuals.

## 1. Introduction

As the global aging process accelerates, the mental health of the elderly has emerged as a central concern within the international public health arena. In 2002, the World Health Organization (WHO) proposed the concept of “active aging” at the Second World Assembly on Ageing. The concept emphasizes that the elderly should be healthy, engaged, and secure throughout their lives. However, this vision faces growing challenges, particularly from mental health issues such as depression. As one of the most common mental disorders among older adults, depression has become a critical barrier to the advancement of active aging initiatives. According to the American Diagnostic and Statistical Manual of Mental Disorders, depression among older adults refers to clinical depression diagnosed in individuals over the age of 60. It not only diminishes satisfaction [1] and quality of life [2] among the elderly but also amplifies the burden on caregivers. Recent studies have documented a rising prevalence of depression among older adults, with 35.1% experiencing depressive symptoms and 13.1% meeting the criteria for major depressive disorder [3,4]. As China is a deeply aging country, the Chinese government pays particular attention to the issue of depression in the elderly. The 14th Five-Year Plan (2021–2025) of the Chinese government promotes active aging as a national strategy, with a particular focus on mental health interventions for the elderly.

As a result of economic progression, population migration, and familial structural evolution, China has seen the emergence of a unique demographic segment termed empty-nest older individuals. According to the data from the Bulletin of the Fifth Sample Survey of the Living Conditions of Urban and Rural Elderly in China, in 2020, the proportion of empty-nest older individuals in China was nearly 60%. In a conventional societal framework, families as the basic social unit play a crucial role in providing emotional support and companionship to older adults. However, modernization has gradually eroded the familial function in elder care, and the absence of close family networks has contributed to increased risks of depression among this population. At the same time, the rapid development of digital finance in China has exerted a profound influence on various aspects of social and economic life, including macroeconomic growth, income distribution, consumption patterns, and everyday convenience [5]. On the one hand, digital finance has the potential to overcome the geographical constraints of traditional finance, reduce economic pressure, and ease financial anxiety, which might help mitigate depression. On the other hand, it may also exacerbate the digital divide, particularly for vulnerable groups such as the elderly, who often face barriers to accessing digital resources due to limited technological literacy. Therefore, this study focuses on China’s empty-nest older individuals and seeks to illuminate the relationship between digital finance and mental health.

The literature relevant to this study primarily falls into two categories: the impact of digital finance on health, and the relationship between Internet use and depression among older adults. Existing studies consistently indicate that digital finance has a positive impact on physical health [6,7,8,9]. However, studies exploring the relationship between digital finance and mental health are still scarce. Liao and Du (2024) [6] found that the development of digital finance reduces the likelihood of depression among residents. Zeng et al. (2025) [10], focusing on the elderly, reported that digital finance can effectively promote their mental health. At the same time, Lin et al. (2024) [11] observed that digital finance has a significant negative effect on the life satisfaction of older adults. It can be observed that research on the health effects of digital finance is still at an early stage, and empirical analyses focusing on the relationship between digital finance and mental health remain particularly limited. Given the limited studies directly linking digital finance to depression among older adults, the relationship between Internet use and depression in the elderly is taken as the second area of research related to this paper. Existing studies have had varied conclusions on the effects of Internet use on depression in older adults. Several studies suggest that Internet use has a positive impact on the mental health of older people, significantly reducing the risk of depression in older age groups [12,13,14]. Studies on Chinese older adults further show that digital inclusion can significantly lower depression scores among those living alone [15]. Online activities are also positively associated with improvements in both physical and mental health [16]. Moreover, digital skills not only promote health but also mitigate the adverse effects of the digital divide [17]. However, Internet use may also have adverse effects [18]. A longer time spent on the internet is associated with a higher degree of depression [19]. These findings indicate that Internet use is a double-edged sword for older adults. Although extensive research has examined the relationship between Internet use and depression among older adults, few studies have explored how digital finance affects the mental health of specific subgroups, such as empty-nest older individuals. Therefore, this paper seeks to address this gap by investigating the impact of digital finance on the depression of empty-nest older individuals.

To assess the impact of digital finance, we employed a fixed-effect OLS model to investigate the likelihood and degree of depression among empty-nest older individuals. Based on the longitudinal data from the China Health and Retirement Longitudinal Study (CHARLS) from 2011 to 2018, our research results indicate that there is a significant negative correlation between digital finance and depression in empty-nest older individuals, as well as the degree of their depression. Moreover, the impact of digital finance on mental health is more pronounced among the empty-nest older individuals in rural areas, those with lower educational attainment, and those without supplementary pensions. To understand the mechanism, we further explored the effects of digital finance on health, participation, and security. The mechanism analysis revealed that digital finance helps enhance the health, social participation, and security of empty-nest older individuals. These findings suggest that digitalization is not an obstacle to active aging. Instead, it enables the elderly to access and benefit from the opportunities and advantages associated with digital finance.

There are three strands to our potential contributions. First, our study enhances the understanding of digital finance by exploring its impact on mental health. While prior studies on digital finance have primarily focused on areas such as economic growth [20], environmental pollution [21], and household finance [22], limited attention has been paid to its impact on mental health. Second, it focuses on empty-nest older individuals, a distinct group that has received limited attention. Although many studies have examined the overall impact of digital technology on older adults, few have specifically focused on empty-nest older individuals who have arisen due to social structural changes. Empty-nest older individuals are more vulnerable than those living with their children and require greater policy and research attention. Third, it introduces the concept of active aging to construct an analytical framework for the impact of digital finance on the mental health of empty-nest older individuals. It also offers a novel perspective for understanding the impact of digitization on vulnerable aging populations and advances the application of active aging within the digital era.

The rest of this paper is organized as follows. Section 2 is the theoretical analysis and research hypothesis. Section 3 presents the data and variables. Section 4 introduces empirical results. Section 5 provides the results of the mechanism analysis. Finally, Section 6 provides the conclusions and policy implications

## 2. Theoretical Analysis and Research Hypothesis

The WHO defines active aging as a dynamic process of optimizing opportunities for health, participation, and security to enhance quality of life as people age. Central to this concept are three fundamental pillars: health, participation, and security. Digital finance, with its ability to transcend spatial constraints, reduce transaction and payment costs, enhance information accessibility, and alleviate financing constraints, exhibits an inherent alignment with the active aging framework. This framework of active aging provides an analytical perspective for understanding how digital finance shapes the mental health of empty-nest older individuals.

The analysis begins with the dimension of social participation. Disengagement theory [23] posits that aging is accompanied by a gradual withdrawal from previously held social roles. This process is particularly prominent among empty-nest older individuals. As they shift from central figures within the family to peripheral roles, many encounter difficulties in role adjustment and identity reconstruction, leading to psychological distress and an increased risk of depression. Activity theory provides a solution by emphasizing that elderly individuals who maintain active social engagement tend to lead happier lives in their later years [24]. However, empty-nest older individuals experience a notable absence of intimate social relationships and support networks compared to non-empty-nest older individuals, which renders them more vulnerable to social isolation [25,26]. In this context, the emergence of digital finance has created new opportunities for social participation. On the one hand, features of digital finance, including mobile payment and QR code transfer, lessen the time and physical demands associated with cash transactions and offline procedures, which in turn support higher levels of daily activity participation among empty-nest older individuals. On the other hand, the social and informational functions embedded in digital finance platforms expand access to activity-related information, partly offsetting the deficits caused by inter-generational separation and the contraction of social networks. Therefore, social participation may constitute a mechanism through which digital finance influences the mental health of empty-nest older individuals.

Moving forward, from the “health” perspective, health plays a pivotal role in determining depression. As individuals age, their health gradually declines, leading to physical deterioration and an elevated risk of depressive symptoms. For empty-nest older individuals, limited accessibility to medical care and the financial burden of medical expenses are major constraints on their health status. Digital finance demonstrates distinct advantages in addressing these challenges. By providing functions such as online registration, payment, and drug purchase, digital finance enables empty-nest older individuals to access medical services at home, reducing travel and waiting times and improving the efficiency of resource allocation, which in turn enhances healthcare accessibility. At the same time, micro-credit and installment payment services in digital finance help alleviate the economic burden of healthcare, enabling empty-nest older individuals to more easily afford medical visits, rehabilitation, and health-related products. Therefore, the improvement of health status may constitute one of the key mechanisms through which digital finance affects the mental health of empty-nest older individuals.

Lastly, from the dimension of “security”, old age is a life stage characterized by a higher vulnerability to poverty. Across the OECD, an average of 14.2% of individuals over the age of 65 live in relative income poverty, defined as having an income of less than half of a country’s average median household disposable income [27]. Existing studies have demonstrated that individual income levels and financial stressors are pivotal factors influencing the psychological well-being of older adults [28,29]. Additionally, economic hardships have been identified as a significant risk factor for depression among this population [30]. Therefore, alleviating economic pressure serves as a critical breakthrough for mitigating depression among empty-nest older individuals. In terms of security, digital finance may influence the mental health of empty-nest older individuals through two pathways. On the one hand, accessible and convenient financial management and savings services enable individuals to accumulate assets and improve liquidity, thereby enhancing their capacity to manage both regular and unforeseen expenses. On the other hand, by expanding financing opportunities for their children, digital finance may enhance household income capacity and thereby create conditions for greater economic support to elderly parents through inter-generational transfers. The overall improvement of personal and household financial conditions enhances the sense of security of empty-nest older individuals. This may constitute one of the important mechanisms through which digital finance affects their mental health.

H: The development of digital finance is significantly negatively correlated with depression among empty-nest older individuals.

## 3. Data, Methods, and Variables

### 3.1. Data

This study examines the potential impact of digital finance on depression among empty-nest older individuals by utilizing two primary datasets. The core data on depression are drawn from CHARLS, covering the period from 2011 to 2018. Supported by Peking University, CHARLS is a nationally representative longitudinal survey targeting Chinese residents aged 45 and above, designed to collect high-quality micro-level data.

CHARLS is particularly suitable for this research due to two key features. First, it offers a large and comprehensive sample. In addition to the 2011 baseline survey (Wave 1), three follow-up waves were conducted in 2013 (Wave 2), 2015 (Wave 3), and 2018 (Wave 4). By 2018, the survey encompassed approximately 19,000 respondents across 150 counties and 450 communities in 28 provinces, municipalities, and autonomous regions. Second, the CHARLS questionnaire is highly detailed, encompassing a wide range of variables, including personal demographics, family financial transfers, health status, medical service utilization, and employment and retirement conditions. These features ensure the provision of sufficient and reliable data for this study. And in this study, the sample comprises respondents aged 60 and above who meet the definition of empty-nest older individuals, namely those living alone or only with a spouse, without co-residing children.

In addition, this study investigates the development of digital finance using the China Digital Financial Inclusion Index, which is jointly developed by the Digital Finance Research Center at Peking University and Ant Financial Group.

### 3.2. Variable

#### 3.2.1. Depression

In the CHARLS, respondents were assessed using the Center for Epidemiological Studies Depression Scale-10 (CES-D10). This scale is a widely recognized tool for measuring the degree of depression globally and has been validated among elderly Chinese populations. CES-D10 consists of 10 items that ask participants to report how often they experienced specific symptoms during the past week, such as difficulty concentrating, feeling depressed, etc. Each item is scored on a scale from 0 to 3, based on the frequency of the symptom, and the total score ranges from 0 to 30. Higher scores indicate a greater degree of depression.

The core explained variable in this study is depression status, a binary indicator derived from the CES-D10 score. Following established practices in the literature, a threshold of 10 is used to identify individuals with depression status [31,32]. Accordingly, respondents with CES-D10 scores greater than 10 are coded as 1, while those scoring 10 or below are coded as 0.

#### 3.2.2. The Degree of Depression

The degree of depression is directly measured by the CES-D10 score, which reflects the cumulative frequency of depressive experiences reported over the past week. A higher score indicates a greater degree of depression and may reveal more subtle variations in mental health than the binary indicator.

#### 3.2.3. Digital Finance

This study employs the Digital Finance Inclusive Finance Index, jointly compiled by the Beijing Digital Finance Research Center and Ant Group Research Institute, as the measurement indicator for digital finance development. The index is structured across three dimensions: (1) breadth of digital finance coverage, (2) depth of digital finance usage, and (3) digitization degree of inclusive finance. This multidimensional framework integrates 33 specific indicators to form a composite evaluation system. Compared with the digital finance indicators included in conventional financial surveys and the regional indices released by research institutions, this index provides broader temporal and spatial coverage, thereby offering a more comprehensive reflection of the evolution of digital finance in China. Moreover, it has been widely adopted in existing studies on China’s digital finance. Building on these strengths, we employ this index to measure the level of digital finance development across cities.

#### 3.2.4. Control Variables

We account for several confounding factors affecting the causal relationship between digital finance and depression, considering both individual and municipal characteristics. At the individual level, variables such as age, marital status, public pension, public medical insurance, cognitive ability, family income, and family size are included to control demographic, socioeconomic, and health-related characteristics that may influence mental health outcomes. At the municipal level, GDP per capita, government scale, and consumption level are incorporated to control for the effect of regional economic development, public service provision capacity, and residents’ consumption capacity. These variables help reduce bias from unobserved factors and better identify the effect of digital finance. Table 1 presents the variable definition and description.

### 3.3. Methods

The dependent variable is a binary measure indicating whether an empty-nest older individual exhibits depression, coded as 1 for presence and 0 for absence. Although a fixed-effect logit model is suitable for a binary explained variable, it excludes individuals whose depression status remains unchanged across survey waves, which may introduce selection bias. To avoid this issue, we adopt a fixed-effect OLS specification in our baseline estimation. The model is as follows:

(1) $${Depression}_{ict}=\beta_{0}+\beta_{1}{DigFin}_{ict}+\delta {Control}_{ict}+\alpha_{i}+\gamma_{t}+\varepsilon_{ict}$$

where ${Depression}_{ict}$ refers to the depression status of respondent *i* in city *c* at time *t*. It is a dummy variable indicating whether the respondent is depressed in the current survey. The key explained variable ${DigFin}_{ict}$ denotes the level of digital finance development in city *c*, where individual *i* is located, at time *t*. Its corresponding coefficient $\beta_{1}$ is the interest of this study, which represents the effect of digital finance on the depression status of the empty-nest older individuals. ${Control}_{ict}$ represents household and city time-varying controls. Standard errors are clustered at the city level. The term $\alpha_{i}$ accounts for an individual fixed effect. In addition, we also added a linear time trend $\gamma_{t}$ to control changes over time. $\beta_{0}$ is the constant term, and $\varepsilon_{ict}$ is the random disturbance term.

Building on this model, we further examine the relationship between digital finance and the degree of depression. In this case, the explained variable is no longer a binary indicator, but rather a count variable ranging from 0 to 30. Considering that the CES-D10 score is a continuous measure of depression, a fixed-effect OLS specification is an appropriate estimation approach and ensures consistency with Model (1). The empirical specification is presented as follows:

(2) $${CED}_{ict}=\beta_{0}+\beta_{1}{DigFin}_{ict}+\delta {Control}_{ict}+\alpha_{i}+\gamma_{t}+\varepsilon_{ict}$$

where ${CED}_{ict}$ is defined as the degree of depression of respondent *i* in city *c* at time *t*, with higher scores indicating more severe depressive symptoms. The coefficient $\beta_{1}$ estimates the influence of digital finance on the degree of depression among empty-nest older individuals. All remaining variables in Model (2) are consistent with those in Model (1).

## 4. Results and Discussions

### 4.1. Descriptive Statistics

Table 2 reports descriptive statistics for the samples. The mean value of depression is 0.30, indicating that approximately 30% of the sample reported experiencing depression during the observation period. The mean value of the degree of depression is 8.09, which is below the commonly used clinical threshold of 10, yet sufficiently close to indicate a potential risk of depression among empty-nest older individuals. For the key explanatory variable, the mean value of digital finance is 148.30, with a standard deviation of 67.02, reflecting a comparatively low level of digital finance development across the sampled regions, alongside substantial variation. Finally, the descriptive statistics of other variables are shown in Table 2, and all fall within reasonable ranges.

### 4.2. Baseline Results

Table 3 reports the estimation results regarding the impact of digital finance on depression among empty-nest older individuals. Column (1) reports the estimation results from Model (1) with individual-level control variables. Based on column (1), column (2) adds control variables at the municipal level. The coefficient of the digital finance in column (2) is −0.003 and is significantly negative at the 1% level, suggesting that the development of digital finance is associated with a lower likelihood of depression among empty-nest older individuals. While the binary depression indicator facilitates an assessment of whether digital finance influences the probability of experiencing depression, it fails to capture variations in the degree of depression. To gain a more detailed understanding, we also employ the degree of depression as the explained variable. Columns (3) and (4) of Table 3 report the estimation results with individual-level control variables and the full set of control variables, respectively. The coefficient of the digital finance in column (4) is −0.028 and is significantly negative at the 1% level, which suggests that digital finance is significantly associated with a lower degree of depression among empty-nest older individuals. It should be noted that the differences in the coefficients of the digital finance in columns (2) and (4) are due to the different measurement scales of the explained variables rather than model specification. While both indicators capture depressive symptoms, one is a binary variable and the other is a continuous score ranging from 0 to 30. Therefore, the coefficients are not directly comparable in magnitude. The interpretation should focus on statistical significance and consistency in effect direction. This finding is consistent with our hypothesis.

Although there is currently no research specifically examining the impact of digital finance on depression among empty-nest older individuals, the findings of this study are consistent with existing research. Liao (2024) [6] identified a significant negative correlation between the development level of regional digital finance and the likelihood of depression among residents.

There are three possible explanations for the mitigating effects of digital finance on the depression of empty-nest older individuals. First, the advancement of digital finance promotes social participation within this group, fulfilling their needs for social interaction and emotional support, which in turn reduces both the likelihood and degree of depression. Second, digital finance enhances access to high-quality digital healthcare services and health-related information, which can mitigate health deterioration. Since physical health is a critical foundation for mental health, improvements in physical health may lead to reduced levels of depression. Third, the development of digital finance may help empty-nest older individuals overcome financial difficulties, thereby reducing the psychological stress associated with economic insecurity.

### 4.3. Robustness Test

#### 4.3.1. Additional Control Variables

To further verify the robustness of the results, this study adds two additional control variables to the baseline regression: the summary score of Activities of Daily Living (ADL) and an indicator for employment in the past year. The ADL summary score captures the physical functioning status of empty-nest older individuals, which is a key factor influencing depression. Controlling for this variable helps to eliminate potential confounding effects caused by physical health conditions. The other variable reflects the respondent’s employment status, which is a proxy for social engagement and economic condition, both of which are closely linked to mental health. Therefore, including these variables in the regression model contributes to a more thorough control of confounding factors and enables a more precise estimation of digital finance on depression among empty-nest older individuals. The regression results with the additional control variables are presented in columns (1) and (2) of Table 4, where column (1) reports whether the respondent is depressed and column (2) measures the degree of depression. The coefficients in columns (1) and (2) are significantly negative, reinforcing the findings of the baseline regression.

#### 4.3.2. Excluding Digitally Advanced Cities

To further address the potential influence of specific cities on the estimation results, we conducted a robustness test by excluding three of the most digitally advanced and influential cities within our sample, namely Hangzhou, Shenzhen, and Shanghai. These cities are characterized by high levels of digital infrastructure, elderly-targeted digital services, and proactive smart eldercare policies. Therefore, their socioeconomic environments may amplify the impact of digital finance on the mental health of the empty-nest older individuals. The regression results after excluding these cities are reported in columns (3) and (4) of Table 4. Column (3) presents the estimates of whether depression is present, while column (4) reports the results for the degree of depression. The estimated coefficients are negative and statistically significant at the 1% level, supporting the robustness of the baseline results.

#### 4.3.3. Alternative Estimation Strategy

To assess the robustness of the baseline estimations, this study replaces the fixed-effect OLS model with the fixed-effect logit model and the fixed-effect ordered logit model. The fixed-effect logit model is employed for whether depression is present, while the fixed-effect ordered logit model is applied to the degree of depression, ensuring alignment between model choice and the measurement scale of the explained variable. The corresponding results are presented in columns (5) and (6) of Table 4. The estimated coefficients remain consistent in both sign and statistical significance, reaffirming the robustness of the primary findings.

### 4.4. Heterogeneity Analysis

#### 4.4.1. Supplementary Pension

Pensions play a critical role in mitigating old-age risks by providing elderly individuals with basic economic security and risk protection. In China’s multi-pillar pension system, the first pillar of basic pension insurance is universal, with broad coverage that guarantees the minimum living needs of the elderly population. The second and third pillars, comprising occupational pensions and commercial pensions, function as supplementary schemes that typically depend on employers or market-based mechanisms to deliver additional income support. Accordingly, the effects of digital finance may vary across groups with different levels of pension protection. To examine this heterogeneity, we divide the sample into two groups based on whether respondents receive occupational or commercial pensions. The results of the heterogeneity analysis are presented in Table 5.

Columns (1) and (3) present estimates for the group with supplementary pensions, while columns (2) and (4) correspond to the group without supplementary pensions. The coefficients of digital finance are significantly negative for empty-nest older individuals without supplementary pensions. However, they are not significant for empty-nest older individuals with supplementary pensions. To further examine the heterogeneity effects, the Chow test was conducted to compare coefficient differences between subgroups. The results show that the *p*-value for the coefficient difference in depression is 0.073, whereas that for the degree of depression is 0.066. These results indicate that the alleviating effect of digital finance on depression is more significant among empty-nest older individuals without supplementary pension insurance. The result is consistent with prior research highlighting the redistributive role of social insurance [33]. In this context, the difference can be explained by the differences in economic security and financial participation capacity between the two groups. Specifically, empty-nest older individuals with supplementary pensions not only have stable pension income but also possess certain financial knowledge and investment skills, which strengthen their risk-coping capacity and thereby weaken the positive effect of digital finance. However, empty-nest older individuals without supplementary pensions lack additional financial protection in old age and have weaker capabilities in wealth management and risk mitigation. To some extent, digital finance compensates for these deficiencies by providing new resources and support, thereby exerting a more significant effect on improving mental health outcomes in this group.

#### 4.4.2. Educational Attainment

Higher educational attainment is generally associated with stronger information acquisition abilities and digital skills, enabling individuals to participate more effectively in both traditional and digital financial activities, while lower-educated individuals often experience difficulties in financial participation. Accordingly, the impact of digital finance on depression among empty-nest older individuals may differ from educational attainment. Based on this, we divided the sample according to whether respondents had attained at least a high school education. The results of the heterogeneity analysis based on educational attainment are presented in Table 6.

Columns (1) and (3) present estimates for empty-nest older individuals with lower educational attainment, while columns (2) and (4) show estimates for empty-nest older individuals with higher educational attainment. The findings reveal a significant negative correlation between digital finance and mental health for empty-nest older individuals with lower educational attainment. Furthermore, the Chow test shows that the coefficient difference between the lower educational attainment group and the higher educational attainment group is significant. The empirical findings indicate that the alleviating effect of digital finance on depression is more pronounced among empty-nest older individuals with lower educational attainment. This difference may be attributed to disparities in financing constraints and informational barriers. Empty-nest older individuals with higher educational attainment usually have easier access to formal credit, greater financial management capacity, and better digital literacy, which reduces their dependence on digital finance. As a result, the incremental benefits of digital finance for their mental health are relatively limited. In contrast, empty-nest older individuals with lower educational attainment are more likely to encounter financing difficulties and lack channels to obtain health information and social support, making them more dependent on digital finance. Consequently, they experience more substantial improvements from the development of digital finance.

#### 4.4.3. Urban and Rural

China has historically allocated more resources to urban areas, leading to marked disparities between urban and rural regions in infrastructure construction and public services. Therefore, when examining the effect of digital finance on the mental health of empty-nest older individuals, it is necessary to account for the urban–rural divide. Based on the residential location of respondents, we divided the sample into two groups: the urban group and the rural group. The results of the heterogeneity analysis are presented in Table 7.

As shown in columns (1) and (2), regarding depression, the coefficient for the rural group is −0.003 and significant at the 1% level, whereas the coefficient for the urban group is −0.002 and significant at the 5% level. However, the *p*-value derived from the Chow test for coefficient differences indicates that the disparity in coefficients across groups is statistically significant. As presented in columns (3) and (4), for the degree of depression, the coefficient for the rural group is −0.036 and significant at the 1% level, while the coefficient for the urban group is −0.017 and significant at the 5% level. The *p*-value for the coefficient difference test is 0.062. Empirical evidence demonstrates that digital finance exerts a significantly stronger effect on alleviating depression among rural empty-nest older individuals than on urban empty-nest older individuals. This disparity may be attributed to differences in the availability of financial services, alternative channels, and the resource base between urban and rural areas. In rural areas, the scarcity of financial institutions and inadequate public resources limits empty-nest older individuals’ access to economic support and information, thereby heightening their mental health vulnerability. Consequently, digital finance provides significant improvements in alleviating depression for them. Compared with rural empty-nest older individuals, urban empty-nest older individuals have greater access to alternative channels; thus, the benefits from digital finance are relatively limited.

## 5. Underlying Mechanisms

### 5.1. Security

The CHARLS questionnaire does not directly measure individuals’ sense of security. However, it includes questions regarding the value of personal cash and savings accounts. Financial assets can provide a buffer against unexpected expenses, health shocks, and unstable income in old age. Having more money in personal cash and savings accounts is associated with a greater sense of security. Therefore, the logarithm of the value of personal cash and savings accounts is employed as a proxy variable for security to investigate the mechanism by which digital finance influences depression among empty-nest older individuals. The estimates are presented in Table 8.

The result in column (1) of Table 8 shows that the coefficient of digital finance is 0.022 and significantly positive at the 1% level. This suggests that digital finance can reduce depression among empty-nest older individuals by increasing security. Digital finance enhances the asset management and financial reserve capacity of empty-nest individuals, while also improving the overall allocation of household economic resources. These individuals can accumulate and increase the value of small savings through convenient investment services, which strengthens their resilience to both daily expenses and unexpected shocks. At the same time, greater access to financing and investment opportunities for their children in the digital financial environment facilitates intergenerational transfers. Improvements in both individual and household financial conditions enhance economic security and perceived control over life, thereby alleviating anxiety and depressive symptoms. Similar results have been documented regarding the positive influence of digitalization on income [34].

### 5.2. Health

As individuals age, their physiological functions progressively decline, resulting in a significant decline in overall health status. This physical degeneration is often accompanied by reduced psychological adaptability, making older adults particularly vulnerable to depression. Under the framework of active aging proposed by the WHO, we further explore whether digital finance can alleviate depression by improving the health status of the elderly. Self-rated health, which captures individuals’ cognitive and subjective evaluations of their physical well-being, is used as a proxy variable in our study for health status. It is scored on a scale of 1–5, with a higher score indicating greater self-rated health. The estimates are presented in Table 8.

The result in column (2) of Table 8 shows that the coefficient of digital finance is 0.003 and significantly positive at the 1% level. This demonstrates that digital finance contributes to improved health outcomes among empty-nest older individuals, thereby enhancing their self-protection capabilities and reducing the risk of depression. Digital finance facilitates a more efficient allocation and utilization of health resources, enabling empty-nest older individuals to access necessary medical and health services within shorter timeframes and under reduced financial pressure. Features such as online registration and the ability to pay in installments lower both physical and economic barriers to healthcare utilization. Additionally, digital platforms offer supplementary services, including health education and medication reminders. By simultaneously improving the affordability and accessibility of healthcare, digital finance contributes to better physical health and a reduced risk of chronic diseases, thereby alleviating depression.

### 5.3. Participation

The WHO emphasizes the importance of active aging and advocates for the participation of older adults in society based on their needs, aspirations, and capabilities. Social participation enables older adults to maintain optimal physical function, decrease mortality risk, enhance cognitive abilities, and improve life satisfaction. However, existing studies suggest that the level of social participation among older adults remains relatively low [35]. With the advancement of digital technologies, opportunities for social interaction have significantly expanded, offering new avenues to enhance social engagement. Therefore, we further examine whether digital finance can alleviate depressive symptoms among empty-nest older individuals by promoting their social participation. In this study, social participation is measured by the number of different types of social activities in which empty-nest older individuals have engaged over the past month. The estimates are presented in Table 8.

The result in column (3) of Table 8 shows that the coefficient of digital finance is 0.026 and significantly positive at the 1% level. This demonstrates that digital finance contributes to improved social participation among empty-nest older individuals, thereby reducing the risk of depression. Digital finance reduces the economic and time barriers to social participation among empty-nest older individuals by providing convenient and low-cost channels for financial transactions and information exchange, while also broadening their access to activity-related information. In particular, digital payment systems minimize the need to carry cash or make repeated visits for in-person transactions, and online community platforms with event notifications enable timely access to cultural, recreational, and volunteer opportunities. These improvements in convenience and information accessibility increase the likelihood of social engagement, thereby alleviating feelings of loneliness and depressive symptoms.

## 6. Conclusions and Policy Implications

### 6.1. Conclusions

In the context of contemporary social development, digitization and population aging have become two prominent trends. Among the elderly population, empty-nest older individuals represent a particularly vulnerable group in the digital era. Enhancing the mental health of this group is crucial for advancing the goals of active aging. When aging intersects with digitization, does it pose a challenge to active aging or does it offer a new opportunity? This study utilizes data from the CHARLS study spanning 2011 to 2018 to investigate the impact of digital finance on the mental health of empty-nest older individuals. Furthermore, we conduct an empirical analysis of the underlying mechanisms through the three dimensions of active aging.

Drawing on data from CHARLS, this study finds that digital finance is positively associated with the mental health of empty-nest older individuals. Specifically, digital finance is linked to a lower likelihood of depression in this group. Further analysis based on CESD-10 scores suggests that digital finance is also related to a reduced probability of empty-nest older individuals reporting higher levels of depression. The analysis further reveals that the impact of digital finance on depression among empty-nest older individuals differs across educational attainment, supplementary pension status, and place of residence. Specifically, the effect is particularly pronounced among empty-nest older individuals with lower educational attainment, without supplementary pensions, and living in rural areas. In addition, our findings reveal that digital finance influences the mental health of empty-nest older individuals through the three dimensions of active aging: health, participation, and security. First, digital finance significantly improves the self-rated health of empty-nest older individuals, thereby enhancing their mental health. Second, it strengthens their perceived sense of security, reducing psychological vulnerability. Third, it promotes social participation, enabling greater social integration and support.

This study has several limitations. First, some relevant confounders, such as direct measures of social support and access to digital education, were unavailable in the CHARLS. Second, no additional subgroup analyses were conducted beyond the reported heterogeneity tests. Third, a bidirectional relationship may exist between digitization and depression, raising the possibility of reverse causality. Fourth, the data cover 2011–2018, so the findings may not fully reflect the rapid technological advances in subsequent years. However, these limitations only underline the need for caution in interpreting or generalizing the results and do not invalidate the conclusions.

### 6.2. Policy Implications

Based on the findings of this study, we offer two policy suggestions to better support the mental health of China’s empty-nest older individuals in the context of rapid digitization and population aging.

First, greater attention should be given to China’s empty-nest older population. As empty-nest families have become the new normal in the context of population aging, preventing and mitigating the adverse consequences associated with this phenomenon should be a key focus of governmental efforts. Therefore, it is essential to accelerate the establishment and improvement of a comprehensive policy framework addressing the needs of empty-nest older individuals, effectively responding to their core economic, social, and health-related concerns and enhancing their overall sense of well-being. For instance, enhancing security by appropriately increasing living subsidies for low-income empty-nest older individuals, improving health by expanding the coverage of community health services and promoting the use of telemedicine and psychological counseling, and fostering social participation by using community activity centers to organize regular interactive activities.

Second, efforts should be made to build an age-friendly digital society. In the digital era, the development of technology should not become a barrier between empty-nest older individuals and society but rather a bridge for them to enjoy the digital dividend and move towards active aging. On the one hand, digital literacy initiatives for older adults should be continuously promoted to improve their adaptability to the digital information ecosystem. Targeted digital skills training should be actively implemented to support empty-nest older individuals in embracing the digital age and enjoying the benefits of smart living. Specifically, it is necessary to expand the supply of educational resources for older adults and encourage senior universities, community schools, and volunteer organizations to offer programs and courses related to digital literacy with a view to strengthening training and assistance for the elderly. On the other hand, digital services should be transformed with a focus on the needs of the elderly, aiming to effectively address the challenges they encounter in digital society and to enhance their overall digital experience. We should continue to promote the development and use of digital products designed for older adults, such as electronic devices and software that are easy to use and meet their daily needs. For example, smartphone apps can offer elder-friendly versions to improve accessibility.

## Figures and Tables

**Table 1:** Variable definition and description.

Variable Type	Variables	Definitions
Explanatory variable	Digital finance	China Digital Financial Inclusion Index
Explained variable	Depression	Depression = 1; non-depression = 0
Explained variable	Degree of depression	CES-D10 score, scored from 0–30, higher scores indicate more severe depression
Individual-level control variables	Age	Age of empty-nest older individuals
	Marital status	Married = 1; unmarried = 0
	Public pension	Public pension participation status Participated = 1; non-participation = 0
	Public medical insurance	Public medical insurance participation status Participated = 1; non-participation = 0
	Cognitive ability	The word recall scores, which are immediate and delayed recall of 10 words, were scored from 0–20, with higher scores indicating greater cognitive ability
	Family income	The logarithm of total family income
	Family size	Number of family members
Municipal-level control variables	GDP	The logarithm of GDP per capita
	Government scale	Local finance general budget expenditure divided by the GDP
	Consumption level	Total retail sales of consumer goods divided by the GDP

**Table 2:** Descriptive statistics.

Variables	N	Mean	SD	Min	Max
Depression	6442	0.30	0.46	0	1
Degree of depression	6442	8.09	6.09	0	30
Digital finance	6442	148.30	67.02	19.53	302.98
Age	6442	69.12	6.14	60	105
Marital status	6442	0.82	0.39	0	1
Public pension	6442	0.55	0.50	0	1
Public medical insurance	6442	0.95	0.22	0	1
Cognitive ability	6442	6.41	3.62	0	20
Family income	6442	8.73	2.22	0	13.60
Family size	6442	2.14	0.91	1	12
GDP	6442	10.57	0.58	8.84	13.06
Government scale	6442	0.18	0.08	0.06	0.70
Consumption level	6442	0.40	0.09	0.03	0.65

**Table 3:** Baseline results.

	(1)	(2)	(3)	(4)
	Depression	Depression	Degree of Depression	Degree of Depression
Digital finance	−0.003 *** (0.001)	−0.003 *** (0.001)	−0.028 *** (0.006)	−0.028 *** (0.007)
Age	−0.034 (0.040)	−0.035 (0.040)	−0.044 (0.463)	−0.042 (0.465)
Marital status	−0.030 (0.043)	−0.030 (0.043)	−0.522 (0.568)	−0.520 (0.567)
Public pension	−0.007 (0.015)	−0.007 (0.015)	−0.169 (0.160)	−0.172 (0.158)
Public medical insurance	0.015 (0.028)	0.014 (0.028)	−0.322 (0.353)	−0.324 (0.353)
Cognitive ability	−0.007 *** (0.002)	−0.007 *** (0.002)	−0.140 *** (0.025)	−0.140 *** (0.025)
Family income	−0.002 (0.004)	−0.002 (0.004)	−0.045 (0.043)	−0.045 (0.044)
Family size	−0.010 (0.010)	−0.012 (0.010)	−0.076 (0.129)	−0.077 (0.125)
GDP		−0.044 (0.062)		0.001 (0.688)
Government scale		−0.137 (0.269)		0.536 (3.925)
Consumption level		−0.049 (0.231)		−0.375 (3.178)
Constant	2.788 (2.620)	3.377 (2.829)	15.074 (30.432)	14.974 (32.806)
Individual FE	√	√	√	√
Time trend	√	√	√	√
Observations	6442	6442	6442	6442
R-squared	0.626	0.626	0.720	0.720

Note: *** *p* < 0.01. Robust standard errors clustered at the city level are reported in parentheses.

**Table 4:** Robustness tests.

	Additional Control Variables		Excluding Digitally Advanced Cities		Alternative Estimation Strategy	
	(1)	(2)	(3)	(4)	(5)	(6)
	Depression	Degree of Depression	Depression	Degree of Depression	Depression	Degree of Depression
Digital finance	−0.003 *** (0.000)	−0.027 *** (0.005)	−0.003 *** (0.001)	−0.029 *** (0.007)	−0.020 *** (0.003)	−0.013 *** (0.002)
Age	−0.041 (0.037)	−0.149 (0.426)	−0.032 (0.041)	0.016 (0.485)	−0.216 (0.287)	−0.013 (0.177)
Marital status	−0.026 (0.033)	−0.456 (0.382)	−0.030 (0.043)	−0.523 (0.568)	−0.239 (0.230)	−0.221 (0.183)
Public pension	−0.007 (0.014)	−0.164 (0.166)	−0.006 (0.015)	−0.140 (0.156)	−0.017 (0.112)	−0.067 (0.074)
Public medical insurance	0.022 (0.028)	−0.195 (0.327)	0.013 (0.028)	−0.353 (0.354)	0.137 (0.221)	−0.098 (0.148)
Cognitive ability	−0.007 *** (0.002)	−0.133 *** (0.026)	−0.007 *** (0.002)	−0.140 *** (0.025)	−0.056 *** (0.018)	−0.064 *** (0.012)
Family income	−0.002 (0.004)	−0.051 (0.041)	−0.002 (0.004)	−0.043 (0.044)	−0.013 (0.027)	−0.019 (0.018)
Family size	−0.014 (0.010)	−0.113 (0.111)	−0.012 (0.010)	−0.077 (0.125)	−0.065 (0.071)	−0.024 (0.050)
GDP	−0.049 (0.050)	−0.097 (0.576)	−0.050 (0.064)	−0.072 (0.715)	−0.494 (0.394)	−0.028 (0.261)
Government scale	−0.155 (0.259)	0.096 (2.974)	−0.124 (0.268)	0.659 (3.919)	−1.312 (2.073)	0.613 (1.416)
Consumption level	−0.089 (0.173)	−1.009 (1.986)	−0.082 (0.233)	−0.814 (3.188)	−0.276 (1.265)	−0.269 (0.900)
ADL	0.043 *** (0.008)	0.743 *** (0.088)	/	/	/	/
Employment	−0.012 (0.018)	−0.274 (0.205)	/	/	/	/
Constant	3.832 (2.522)	23.065 (28.916)	3.219 (2.965)	12.119 (34.325)	/	/
Individual FE	√	√	√	√	√	√
Time trend	√	√	√	√	√	√
Observations	6429	6429	6394	6394	2225	6085
R-squared	0.629	0.726	0.626	0.722	/	/

Note: *** *p* < 0.01. Columns (1)–(4) report robust standard errors clustered at the city level, and Columns (5)–(6) report conventional robust standard errors.

**Table 5:** Heterogeneity effects of supplementary pensions.

	Depression		Degree of Depression	
	(1)	(2)	(3)	(4)
	With Supplementary Pension	Without Supplementary Pension	With Supplementary Pension	Without Supplementary Pension
Digital finance	−0.001 (0.001)	−0.003 *** (0.001)	−0.013 (0.012)	−0.032 *** (0.008)
Age	0.042 (0.045)	−0.072 (0.058)	0.474 (0.612)	−0.125 (0.625)
Marital status	−0.039 (0.078)	−0.048 (0.049)	−1.113 (0.890)	−0.614 (0.666)
Public pension	−0.019 (0.045)	0.005 (0.019)	−0.021 (0.460)	0.010 (0.213)
Public medical insurance	−0.154 *** (0.048)	0.041 (0.035)	−1.745 ** (0.712)	−0.348 (0.435)
Cognitive ability	−0.007 * (0.003)	−0.007 *** (0.002)	−0.107 *** (0.036)	−0.140 *** (0.030)
Family income	−0.004 (0.014)	−0.002 (0.005)	−0.134 (0.175)	−0.062 (0.051)
Family size	0.005 (0.019)	−0.014 (0.012)	0.242 (0.242)	−0.172 (0.158)
GDP	−0.034 (0.079)	−0.026 (0.075)	0.053 (1.006)	−0.063 (0.803)
Government scale	−0.329 (0.435)	−0.160 (0.355)	−3.205 (3.984)	0.257 (5.487)
Consumption level	0.264 (0.291)	−0.030 (0.300)	0.953 (3.489)	0.643 (3.890)
Constant	−1.959 (3.074)	5.653 (4.080)	−20.941 (43.416)	22.011 (43.763)
Individual FE	√	√	√	√
Time trend	√	√	√	√
Observations	1317	4632	1317	4632
R-squared	0.601	0.625	0.725	0.713
*p*-value for the coefficient difference	0.073		0.066	

Note: *** *p* < 0.01, ** *p* < 0.05, * *p* < 0.1. Robust standard errors clustered at the city level are reported in parentheses.

**Table 6:** Heterogeneity effects of educational attainment.

	Depression		Degree of Depression	
	(1)	(2)	(3)	(4)
	Lower Educational Attainment	Higher Educational Attainment	Lower Educational Attainment	Higher Educational Attainment
Digital finance	−0.003 *** (0.001)	−0.000 (0.001)	−0.033 *** (0.007)	0.007 (0.013)
Age	−0.029 (0.045)	−0.062 (0.066)	−0.047 (0.498)	0.269 (0.855)
Marital status	−0.018 (0.045)	−0.170 ** (0.082)	−0.369 (0.580)	−2.163 (1.720)
Public pension	0.000 (0.016)	−0.046 (0.053)	−0.077 (0.167)	−0.314 (0.533)
Public medical insurance	0.017 (0.030)	−0.040 (0.057)	−0.376 (0.369)	−0.294 (1.265)
Cognitive ability	−0.008 *** (0.002)	−0.006 (0.005)	−0.144 *** (0.026)	−0.113 * (0.061)
Family income	−0.001 (0.005)	−0.005 (0.007)	−0.048 (0.047)	−0.023 (0.095)
Family size	−0.011 (0.010)	−0.011 (0.021)	−0.086 (0.131)	−0.027 (0.289)
GDP	−0.048 (0.068)	−0.010 (0.093)	−0.159 (0.740)	1.418 (1.272)
Government scale	−0.245 (0.315)	0.794 * (0.440)	0.060 (4.584)	6.570 (5.120)
Consumption level	−0.013 (0.262)	−0.370 (0.309)	0.015 (3.567)	−3.888 (4.487)
Constant	3.021 (3.190)	4.758 (4.462)	17.414 (35.036)	−24.003 (58.789)
Individual FE	√	√	√	√
Time trend	√	√	√	√
Observations	5829	613	5829	613
R-squared	0.618	0.694	0.715	0.735
*p*-value for the coefficient difference	0.034		0.005	

Note: *** *p* < 0.01, ** *p* < 0.05, * *p* < 0.1. Robust standard errors clustered at the city level are reported in parentheses.

**Table 7:** Heterogeneity effects of urban and rural areas.

	Depression		Degree of Depression	
	(1)	(2)	(3)	(4)
	Rural	Urban	Rural	Urban
Digital finance	−0.003 *** (0.001)	−0.002 ** (0.001)	−0.036 *** (0.008)	−0.017 ** (0.008)
Age	−0.029 (0.068)	−0.044 (0.039)	0.045 (0.767)	−0.198 (0.550)
Marital status	−0.007 (0.052)	−0.065 (0.069)	−0.080 (0.714)	−1.174 (0.858)
Public pension	0.008 (0.021)	−0.015 (0.022)	0.008 (0.216)	−0.226 (0.236)
Public medical insurance	0.035 (0.041)	−0.027 (0.039)	−0.188 (0.471)	−0.671 (0.517)
Cognitive ability	−0.006 * (0.003)	−0.010 *** (0.003)	−0.119 *** (0.034)	−0.175 *** (0.033)
Family income	0.004 (0.006)	−0.007 (0.006)	−0.045 (0.063)	−0.041 (0.065)
Family size	−0.009 (0.014)	−0.019 (0.014)	−0.053 (0.147)	−0.167 (0.209)
GDP	0.005 (0.094)	−0.112 * (0.062)	0.260 (1.025)	−0.237 (0.624)
Government scale	−0.280 (0.436)	0.002 (0.243)	−0.773 (6.148)	1.465 (3.232)
Consumption level	−0.073 (0.329)	−0.009 (0.230)	−2.249 (4.398)	3.158 (2.855)
Constant	2.448 (4.833)	4.707 * (2.759)	7.849 (53.314)	26.042 (37.272)
Individual FE	√	√	√	√
Time trend	√	√	√	√
Observations	3867	2575	3867	2575
R-squared	0.616	0.630	0.714	0.714
*p*-value for the coefficient difference	0.034		0.062	

Note: *** *p* < 0.01, ** *p* < 0.05, * *p* < 0.1. Robust standard errors clustered at the city level are reported in parentheses.

**Table 8:** Underlying mechanism.

	(1)	(2)	(3)
	Security	Health	Participation
Digital Finance	0.022 *** (0.005)	0.003 *** (0.001)	0.026 *** (0.002)
Control variable	Yes	Yes	Yes
Individual FE	√	√	√
Time trend	√	√	√
Observations	5585	6440	6442
R-squared	0.700	0.667	0.644

Note: *** *p* < 0.01. Robust standard errors clustered at the city level are reported in parentheses.

## Data Availability

The original data are available at https://charls.pku.edu.cn/.
